# 948. Breathing Life into Respiratory Treatment Compliance: Removing Barriers to Improve Success in Respiratory Medication Administration

**DOI:** 10.1093/jbcr/irag033.515

**Published:** 2026-04-06

**Authors:** Demetria Davis, Wilbur Kennedy, Cynthia Alexander, Carey K Lamphier, Jasmin Mercedes, Laura S Johnson, Jenny Han, Lauren B Nosanov

**Affiliations:** Grady Memorial Hospital, Atlanta, GA; Grady Health System, Atlanta, GA; Grady Health System, Atlanta, GA; Grady Memorial Hospital, Atlanta, GA; Grady Memorial Hospital, Atlanta, GA; Grady Memorial Hospital / Emory School of Medicine / Morehouse School of Medicine, Atlanta, GA; Grady Health System, Atlanta, GA; Emory University School of Medicine, Atlanta, GA

## Abstract

**Introduction:**

Administration of medications by Respiratory Therapists (RT) is crucial for the prevention, treatment, and reduction of respiratory failure in burn injured patients. Patients are at risk of elevated respiratory complications due to direct lung injury secondary to smoke and fume inhalation, infection, volume overload, and overall inflammatory response. Through tracking compliance with respiratory medication administration and identification of barriers to treatment administration we sought to establish an action plan for process improvement.

**Methods:**

Data were obtained from medication administration reports between 1/2024-4/2025. All ordered nebulized and metered-dose inhaler (MDI) treatments for all burn patients, including those who were critically ill, were included in the study. Data were obtained through the Pharmacy Informational Technology and Clinical Quality Analytics departments. After investigation on barriers to respiratory medication administration (characterized as modifiable and non-modifiable), workflow was revised to remove modifiable barriers to increase adherence. Education was provided, and adherence was audited, with additional chart review and case queries performed as needed. Results were reported to the Burn Unit leadership during quality meetings and used to generate a missed treatment protocol.

**Results:**

There were 4048 treatments scheduled during the study period. Month-to-month compliance ranged from 65.2-97.5%, with 131 patients missing 427 treatments. Reasons identified for missing respiratory medication treatments included: other (151, 35.4%), patient/family refused (145, 34.0%), patient not available (49, 11.5%), medication not available (34, 8.0%), order parameters not met (25, 5.9%), and contraindicated (23, 5.4%). "Other” reasons included unstable vital signs and patient/family refusal. All RT staff were educated on the standard protocol algorithm to resolve modifiable barriers such as unavailable medications and providing patient centered education to family members to explain medication importance (Fig. 1).

**Conclusions:**

Most modifiable barriers that were removed to enhance respiratory medication administration were medication availability, patient or family refusal, and patient accessibility due to other services providing care to the patient.

**Applicability of Research to Practice:**

By first analyzing specific barriers to treatment compliance, departments can implement targeted strategies tailored to their unique challenges. Categorizing these barriers as modifiable or non-modifiable enables prioritization of actionable areas and develop effective, protocol-driven solutions. This structured approach offers a scalable model for sustainable, hospital-wide quality improvement.

**Funding for the study:**

N/A.

